# Fluid status evaluation by inferior vena cava diameter and bioimpedance spectroscopy in pediatric chronic hemodialysis

**DOI:** 10.1186/s12882-017-0793-1

**Published:** 2017-12-28

**Authors:** Xavier Torterüe, Laurène Dehoux, Marie-Alice Macher, Olivier Niel, Thérésa Kwon, Georges Deschênes, Julien Hogan

**Affiliations:** Department of Paediatric Nephrology and Hemodialysis, Hôpital Robert Debré, APHP, 48 boulevard Sérurier 75019, 19 Paris Cedex, France

**Keywords:** Inferior vena cava, Bioimpedance, Hemodialysis, Blood pressure, Children, Dry weight

## Abstract

**Background:**

Evaluation of patient’s dry weight remains challenging in chronic hemodialysis (HD) especially in children. Inferior Vena Cava (IVC) measurement was reported useful to assess fluid overload both in adults and children.

**Methods:**

We performed a monocentric prospective study to evaluate the relation between predialytic IVC diameter measurements and hydration status evaluated by physicians and bioimpedance spectroscopy (BIS) and between IVC measurements and persistent hypertension.

**Results:**

Forty-eight HD sessions in 16 patients were analyzed. According to physicians, patients were overhydrated in 84.5% of dialysis sessions, 20.8% according to BIS, and 0%, 4.1% and 20.8% according to IVC inspiratory, expiratory and collapsibility index reference curves respectively. There was no correlation between relative overhydration evaluated by BIS and IVC measurements z-scores (*p* = 0.20). Patients whose blood pressure normalized after HD had a more dilated maximal IVC diameter before dialysis session than patients with persistent hypertension (median − 0.07SD [−0.8; 0.88] versus −1.61SD [−2.18; −0.74] (*p* = 0.03)) with an optimal cut-off of −0.5 SD.

**Conclusions:**

In our study, IVC measurement is not reliable to assess fluid overload in children on HD and was not correlated with extracellular fluid volume assessed by BIS measurements. However, IVC measurements might be of interest in differentiating volume-dependant hypertension from volume-independant hypertension.

**Electronic supplementary material:**

The online version of this article (10.1186/s12882-017-0793-1) contains supplementary material, which is available to authorized users.

## Background

Hydration status evaluation is one of the major issues in children on chronic hemodialysis (HD). Acute overhydration can lead to acute pulmonary oedema and hypertension while chronic overhydration is a well-known factor of cardiovascular morbidity and mortality both in adults and children [1, 2]. Intradialytic underhydration induces headache, abdominal pain, muscle cramps, hypotension and has been shown to increase the risk of brain and heart injury [3, 4]. Postdialytic underhydration exposes the patient to an increased interdialytic weight gain which has been found associated with left ventricular hypertrophy and cardiovascular death [5].

Many methods exist to complete the clinical evaluation of the hydration status: cardiothoracic index based on chest X-ray evaluation, Inferior Vena Cava (IVC) diameter evaluated by ultrasound, biomarkers like Brain Natriuretic Peptide, Bioimpedance Spectroscopy (BIS), plasmatic volume variation monitoring [6] and recently lung ultrasound [7]. Among adults, a strict management of the hydration status based on BIS evaluation was found effective in decreasing Left Ventricular Mass Index and all-cause and cardiovascular mortalities [8, 9]. Based on these results, BIS is now widely recognized as the method of reference for hydration status assessment among adult patients on chronic HD.

Measurement of IVC diameter by echocardiography is a rapid, non-invasive and relatively easy-to-use method to estimate the central venous pressure (CVP) [10, 11]. Among adults, a minimal IVC diameter inferior to 8 mm/m^2^ is associated to a low CVP less than 3 mmHg and a minimal IVC diameter superior to 11.5 mm/m^2^ is associated to a high CVP above 7 mmHg [12–14]. Nephrologists can use it easily without the help of a cardiologist [15]. Many studies showed that a dilatation of IVC is associated with an overhydration in adult population on hemodialysis and a collapsed IVC might be used to define dry weight [13, 16]. Brennan et al. showed that a low predialytic IVC Collapsibility Index (IVCCI) was associated with intradialytic adverse events [14]. However, only few pediatric studies evaluated this technique among children treated with HD and showed a decreased IVC diameter and an increased IVCCI after dialysis [17–19]. But these studies were performed before the American Society of Echocardiography published reference curves for maximal IVC diameter, minimal IVC diameter and IVC collapsibility index indexed to body surface area based on repeated measurements in a healthy pediatric population [20]. Thus, studies assessing the association between IVC measurements and fluid overload, dialysis tolerance or cardiovascular events, are lacking in children.

In this single-center prospective study, we aim to (I) evaluate the ability of predialysis IVC measurements to determine patients’ fluid status before dialysis session and to guide dry weight assessment, (II) to evaluate the usefulness of IVC measurement to predict the tolerance of the HD session and (III) to study the IVC measurements as a marker of intravascular volume and blood pressure in HD pediatric patients.

## Methods

### *Patients’ characteristics* and c*linical evaluation of hydratation status:*

All prevalent patients on chronic HD in our center between March and April 2015 were included. We excluded one patient because of vascular abnormalities (absence of IVC) and one because of the absence of consent. Informed consent was obtained from the parents or guardian of the patients prior to the study after the local ethics committee approved the research protocol.

Systolic blood pressure (SBP), diastolic blood pressure (DBP), heart rate (HR) and weight were measured just before connection and after dialysis session according to the unit practices. The blood pressure was measured in supine position with an automated device and an adapted cuff size. Hypertension was defined as superior to the 95th percentile for age, height and sex according to the Pediatric BP Task Force Report [21]. Blood pressure is reported in SBP index (SBP divided by SBP 95th percentile) and DBP index (DBP divided by DBP 95th percentile). The patients’ clinical dry weight was evaluated by a physician using clinical history, blood pressure, cardiothoracic ratio on chest X-ray, hematocrit and protidemia, tolerance of prior hemodialysis sessions and interdialytic weight gain. Dry weight was regularly decreased until blood pressure is controlled or dialysis session tolerance precludes further decrease. Clinical evaluation of overhydration was calculated as follow: (Pre-dialysis weight – Dry weight)/Dry weight and express in percents. Total ultrafiltration, ultrafiltration rate (in mL per minute) and hemodialysis duration were chosen according to this clinical assessment. The physician was unaware of IVC and BIS measurements. Hemodialysis session tolerance evaluation included hypotension, headache or abdominal pain during the session. In case of intolerance, SBP, DBP, HR, total ultrafiltration, hourly ultrafiltration rate and total amount of volemic expansion (if needed) were recorded. Patients received 3 or 4 hemodialysis sessions per week of four hours each. At the time of the study, sodium profiling was not routinely performed in our center and ultrafiltration was not guided by relative blood volume modification. Inclusions were made indifferently during midweek sessions or after the weekend.

### Echocardiographic measurement:

The echocardiographic measurements were performed at the bedside by a nephrologist of the unit with a patient in supine position for at least 5 min just before the connection and the beginning of the dialysis session and one hour after the end of the session to allow vascular refilling after dialysis. The IVC diameter was measured at end-expiration (maximal diameter) and at end-inspiration (minimal diameter) at the entry of the hepatic veins as recommended by the American Society of Echocardiography guidelines and performed in the study by Kutty et al. We chose to make the measurements with the two-dimensional technique, B-mode, that had the spatial orientation advantages we lose in M-mode [10, 20]. IVCCI was calculated as IVCCI = (maximal diameter – minimal diameter)/maximal diameter. The different measurements were then reported on the reference curves indexed on BSA. Maximal or minimal IVC diameter superior to +2SD and IVCCI inferior to -2SD were considered as a dilatation of IVC. Maximal or minimal IVC diameter inferior to -2SD and IVCCI superior to +2SD were considered as a collapsed IVC [20]. Ultrasound studies were performed on an Philips ATL® HDI 3500.

### Assessment of hydration status by bioimpedance spectroscopy

Body Composition Monitor (BCM, Fresenius Medical Care®) using multifrequency BIS was used to assess the hydration status. At least two measures were realized at the same time of the echocardiographic measurement, before connection, at the bedside, and after five minutes of supine position. The BCM device is a non-invasive method to estimate the intracellular and extracellular water volume by the use of bioimpedance spectroscopy at 50 frequencies (5 kHz to 1000 MHz). The relative overhydration (rel.OH), hydration normalized to extracellular water, allows the comparison between the patients regardless of their weights, heights, sex or age. The normal hydration status was defined by a rel.OH from −7 to 7%, corresponding to the 10th and 90th percentiles of a healthy population. A moderate overhydration was defined by a rel.OH from +7 to +15% and a severe overhydration by a rel.OH > +15% [2, 22]. These norms were used in studies in adult [23] and in paediatric [24] populations.

### Statistical analysis

Dichotomous variables are given in percent and continuous variables as median and interquartiles (IQ). We tested for correlation between the results of the different methods used to assess the hydration status by calculating Pearson correlation coefficient. Logistic regression models were used to study factors associated with the tolerance of the dialysis sessions. We first performed univariable logistic regressions on all the variables to determine which ones to include in our final model. All variables with a *P*-value less than 0.2 were included in the multivariable logistic regression models.

All tests were performed at an α-risk value of 0.05. Statistical analysis was performed with SAS 9.2.

## Results

### Patients’ characteristics

Sixteen patients were included (13 boys and three girls). The median age was 14.3 years old [9.9–15.5]. The median time from the start of hemodialysis was 19 months [9.5–21.8]. The median Body Mass Index was 17.8 kg/m^2^ [16.1–19.4]. Five patients were treated for hypertension, four required one anti-hypertensive drug and one required three. Five patients had bilateral nephrectomy before the study period and only three patients had urine output greater than 0.5 ml.kg^−1^.h-^1^. Most of the patients had three dialysis sessions a week and only three patients had four sessions a week. Vascular access was central venous catheter in six patients and A-V fistula in 10. The median urea Kt/V was 1.34 [1.2–1.9].

### Hydration status assessment

In our cohort, patients were considered overhydrated in 84.5% of dialysis sessions by clinical assessment before dialysis session. The median ultrafiltration volume was 35.4 ml/kg/session [15.6–51.4] with a loss of 3,4% of predialysis body weight by session [1.3–4.3].

Ninety-six echocardiographic measurements were performed. There was a significant decrease of IVC diameter after hemodialysis sessions considering both the minimal (median − 1.88 mm [−2.88; −0.88] (*p* = 0.0004)), and the maximal (median−3.17 mm [−4.62; −1.73] (*p* < 0,0001)) IVC diameter. We also found a median IVCCI increase of 0,10 mm [0.03; 0.17] (*p* = 0,006). There was a good correlation between IVC maximal and minimal diameter measurement (Pearson correlation coefficient = 0.77). Measurements were reported on the reference curves (Additional file 1: Figure 1 a-c). There was no statistically significant correlation between overhydration estimated by clinical evaluation and predialysis IVC maximal and minimal diameters and IVCCI z-scores.

**Fig. 1:**
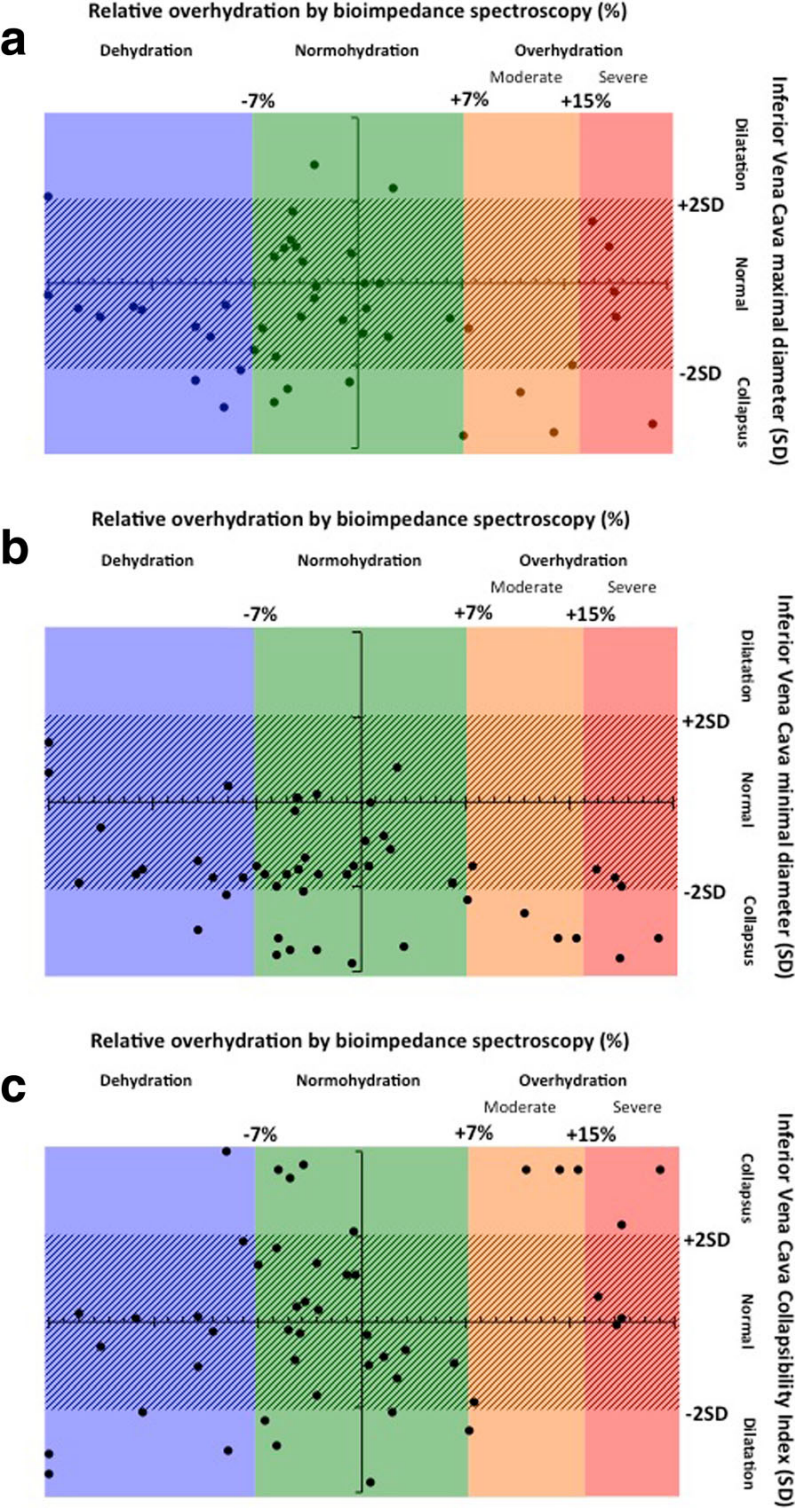
Inferior Vena Cava measurement by hydration status evaluated by bioimpedance spectroscopy (**a**) maximal diameter, (**b**) minimal diameter, (**c**) collapsibility index

A total of 48 BIS measurements were analyzed (three per patient). Mean rel.OH was −3.4% (range − 7.25 to +2.52) and 52.1% of patients were found to be normohydrated (rel.OH -7 to +7%) whereas 25% were found dehydrated (rel.OH < −7%), 10.4% moderately overhydrated (7% < rel.OH < 15%) and 10.4% severely overhydrated (rel.OH >15%). There was no statistically significant correlation between overhydration estimated by clinical evaluation and BIS evaluation (Pearson correlation coefficient = 0.24, *p* = 0.10). Considering BIS evaluation as reliable, 46% of the sessions were correctly classified for hydration status based on IVC diameters and 48% based on predialysis IVCCI (Fig. 1a-c). No significant association was found between the relative overhydration assessed by BIS and predialysis IVC measurements z-scores. Even the best echographic marker, namely IVCCI, had a low sensitivity of 40% and a specificity of 84%.

To test whether those results could be explained by inappropriate reference curves, we tested the correlation between the echographic measurements normalized for body surface area and the relative overhydration estimated by BIS and found no significant correlation.

### Dialysis tolerance

Eleven dialysis sessions (23%) were associated with symptoms of intolerance. Two patients (4%) had abdominal pain, nine (19%) had sudden drop of blood pressure with associated tachycardia. There was a decrease of 30% of systolic blood pressure (range 23 to 35%) and 33.4% of diastolic blood pressure (range 23.4 to 38.4%). Nine patients had tachycardia with a median increase of 17.2% of heart rate (range 12.9 to 20.5%). When intolerance symptoms occurred, all patients underwent passive leg rising and ultrafiltration stop. Three received volume expansion of isotonic saline solution.

Patients’ age, blood pressure at start of dialysis, hydration status evaluated by BIS and echocardiographic measurements were not found to be associated with the tolerance of dialysis sessions (Table 1). There was a trend towards an association between ultrafiltration rate and dialysis intolerance OR 1,17 [0, 98–1, 40] (*p* = 0, 08).

**Table 1:** Odds ratio of dialysis intolerance (Univariate and Multivariate logistic regression)

		Relation with dialysis intolerance (OR)			
		Univariate	p	Multivariate	p
High blood pressure		0,86	0,87		
Age		0,94	0,47		
BIS hydration	<−7%	0,33	0,55		
	> + 7%	0,44			
Clinical overhydration		1,2	0,12	0,91 [0,65–1,28]	0,59
Hourly Ultrafiltration		1,13	0,04	1,17 [0,98–1,40]	0,08
IVCCI	<2DS	1,5	0,82		
	>2DS	0,67			
IVCCI/BSA		1,37	0,53		
IVCmin	<2DS	0,69	0,68		
	>2DS	–			
IVCmin/BSA		0,99	0,97		
IVCmax	<2DS	1,55	0,89		
	>2DS	Not converged			
IVCmax/BSA		0,97	0,75		

*OR* Odds Ratio

*BIS* Bioimpedance Spectroscopy

*IVCCI* Inferior Vena Cava Collapsibility index

*IVCmin* Inferior Vena Cava minimal diameter in inspiration

*IVCmax* Inferior Vena Cava maximal diameter in expiration

*BSA* Body Surface Area

### Study of blood pressure

**Fig. 2:**
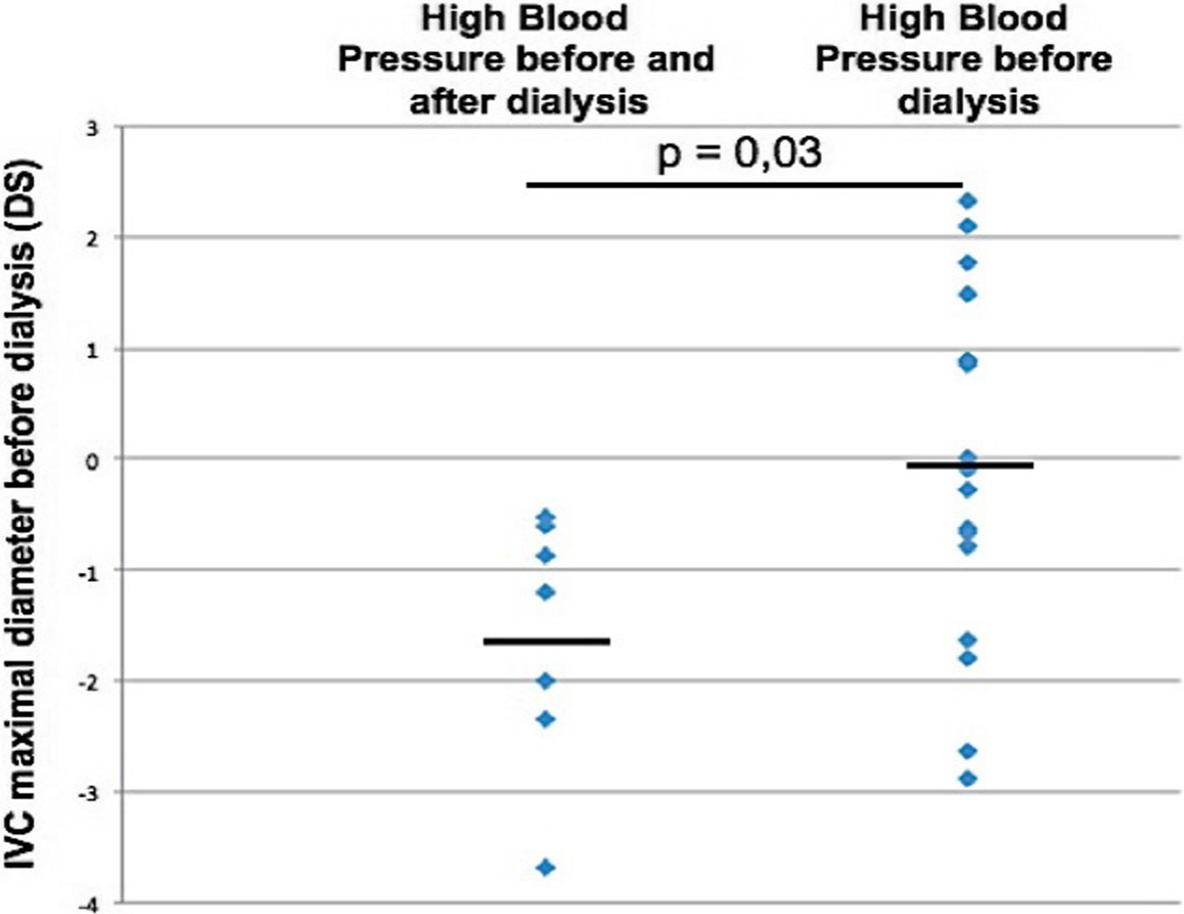
IVC maximal diameter measurement before dialysis session reported on references curves of the American Society of Echocardiography in patients with persistence of high blood pressure after dialysis session and patients with normalization of high blood pressure

Seventeen sessions were associated with predialysis high blood pressure (SBP index 0.98 [0.97; 1.08] and DBP index 1.06 [1.02; 1.13] that normalized after HD session (SBP index (0.88 [0.83; 0.91] and DBP index 0.83 [0.77; 0.92]). After seven sessions, some patients had remaining high blood pressure (SBP index 1,09 [1,06; 1,10] and DBP index 1,14 [1,00; 1,17] before HD; and SBP index 1.02 [0.97; 1.06] and DBP index 1.03 [0.91; 1.04]). We found no difference of hydration status assessed by BIS before hemodialysis between patient presenting high blood pressure and those with normal pressure (*p* = 0,28), and between patients with persistent hypertension and patients who normalized their blood pressure after the session (*p* = 0,30). None of the patients with persistent high blood pressure had bilateral nephrectomy. Two of the twelve patients had predialytic high blood pressure despite antihypertensive therapy. None of these patients had postdialytic hypertension. Ultrafiltration during the session was significantly higher for patients with persistent high blood pressure than for those whose blood pressure normalized (median 98 ml/kg [49; 105] versus 34 ml/kg [22; 44] (*p* = 0.001)). Patients whose blood pressure normalized after HD had a more dilated maximal IVC diameter before dialysis than those with persistent hypertension (median − 0.07SD [−0.8; 0.88] versus −1.61DS [−2.18; −0.74] (*p* = 0.03)) (Fig. 2). A cutoff of IVC diameter inferior to 0,5SD was able to discriminate patients with volume-independant hypertension from those with volume-dependant hypertension with a specificity of 100% and a sensibility of 59%.

## Discussion

In this cohort of 16 children on chronic hemodialysis, we do not find any correlation between IVC diameter measured by echography and hydration status evaluated by bioimpedance spectroscopy. But we found an interesting association between persistent hypertension and end-expiratory IVC diameter.

Onofriescu et al. showed that bioimpedance-guided management improves survival of patient in chronic dialysis in comparison with clinical assessment [9]. That is consistent with the absence of a correlation between clinical assessment and BIS assessment of the dry weight in our study and in an adult study [25]. This underlines the difficulty to assess hydration status clinically. However, one major limitation of BIS is its inability to assess intravascular volume.

Inferior vena cava maximal diameter is known to correlate with central venous pressure (CVP) among critically ill and healthy adult patient [10, 11]. The same correlation has been found between IVC collapsibility index and CVP in a paediatric population [26]. Based on these physiological data, studies in an adult population demonstrated that echocardiography may be used to determine the dry weight [13, 14, 16]. Moreover, Chang et al. showed that echocardiography is a useful tool to improve architecture and cardiac function after long term echocardiographic dry weight management [27].

In our study, we found significant modifications of IVC diameter between pre and postdialysis measurements, which is consistent with previous studies. Indeed, three studies demonstrated a decrease of IVC diameter after dialysis session in children [17–19]. Haciomeroglu et al. found IVCCI to be significantly lower in nine dialysis patients before hemodialysis sessions than in healthy patient. This difference did not remain significant after dialysis sessions [19].

In 2000, Dietel et al. published referent curves for IVC maximal diameter [28] based on ultrasound measurements in 206 pediatric patients. There was a lack of referent curves for the other parameters (IVC minimal diameter and IVCCI) until Kutty et al. study [20]. Dietel et al. found an inverse correlation between IVC maximal diameter and resistance measured by BIS. But they did not study the relationship between resistance and hydration status in children on chronic hemodialysis even if they report resistance on their resistance curves.

However, we found no correlation between hydration status evaluated by IVC measurement based on the reference curves from Kutty et al. and the hydration status assessed by BIS. Thus, the dilatation or the collapsus of IVC does not seem to be a good reflection of hydration status. Allinovi et al. found similar results when comparing 22 IVC measurements in 13 patients on peritoneal and hemodialysis to the hydration status evaluated by BIS [7]. These might be explained by the repartition of extravascular volume between the interstitial and the intravascular compartments. Despite being a good marker of CVP, IVC measurements are poor markers of extracellular hydration. The modification of IVC diameter and IVCCI during dialysis session may thus only be a marker of intravascular volume depletion.

Our second objective was to assess whether IVC measurements could help predict dialysis tolerance. The only factor in our study which seems to be associated with perdialytic complications is a high ultrafiltration rate. That is consistent with previous pediatric studies [29, 30]. We did not confirm the association between high IVCCI before dialysis and perdialytic complications that were reported by Brennan et al. [14]. That might be explained by differences in clinical dry weight assessment.

We determined ultrafiltration volume on the clinical parameters and per dialytic complications during previous sessions. The underestimation of dry weight by clinical assessment found in our study might be due to the use of high blood pressure as a marker of overhydration although dialysis patients are known to frequently present fluid independent hypertension [24]. That might explain the high rate of dialysis complications in our study (23%). Although clinical examination will always play a role, high blood pressure should be interpreted carefully in hydration status evaluation. In that regards, IVC measurements can be helpful in detecting patients whose hypertension is at least partially volume-dependant and will benefit from volume depletion.

To our knowledge, the present study is the first one to compare hydration status assessment by echographic measures of IVC reported on referent curves to BIS evaluation in a cohort of children on hemodialysis. Our study has several limitations. First, the small population size due to the scarcity of ESRD in children and the monocentric design of the study. The second is the lack of “gold standard” evaluation of hydration status. Indeed deuterium dilution is not available in clinical practice. Although BIS measurements and measurements by dilution techniques correlates, BIS have been reported to be less precise than dilution techniques [15]. The third is the timing of postdialysis inferior vena cava evaluation. The complete refilling of intravascular compartments occurs within 2 to 3 h after the end of the dialysis session [12, 16]. Therefore, post dialysis measurements should be performed 2 h after dialysis. However, to preserve outpatient quality of life and to be as close as possible to what could be done if this method was used in everyday practice, we chose to perform IVC measurements one hour after the end of the session. This timing was also chosen in the three previous paediatric studies on IVC evaluation [17–19, 28]. This enables us to compare our results to the literature. Considering blood pressure assessment, although dry weight was carefully reassessed at each session and decreased as much as possible, we cannot completely rule out that some of the patients with persistent high blood pressure after dialysis remain volume overload. Finally, sodium profiling and blood-volume monitoring were not currently used at the time of the study. Indeed, relative blood volume decline is correlated with perdialysis adverse event [30, 31] and the blood-volume monitoring allows a real-time control of ultrafiltration. Sodium profiling allows the variation of the ultrafiltration rate during the hemodialysis session and the decrease of intradialytic hypotension [32]. The absence of blood-volume monitoring might explain the correlation between perdialysis adverse event and the rate of ultrafiltration in our cohort.

## Conclusion

Our study indicates that predialityc measurement of IVC diameter is not sufficient to assess hydration status but can predict volume-dependant high blood pressure in children on chronic HD. Thus, other methods are needed and the impact of the use of BIS on acute and chronic haemodialysis complications has to be evaluated in future studies.

## Additional file

Additional file 1: IVC measurement before and one hour after dialysis session reported reference curves of the American Society of Echocardiography: maximal and minimal diameter and collapsibility index. (JPEG 101 kb)

## Abbreviations

BIS: *bioimpedance spectroscopy*
BMI: *Body mass index*
BSA: *Body surface area*
CVP: *Central venous pressure*
DBP: *Diastolic blood pressure*
HD: *Hemodialysis*
HR: *Heart rate*
IVC: *Inferior vena cava*
IVCCI: *Inferior vena cava collapsibility index*
SBP: *Systolic blood pressure*
