# Supplementary figures and images for: Fluid status evaluation by inferior vena cava diameter and bioimpedance spectroscopy in pediatric chronic hemodialysis
Source: BMC Nephrol. 2017 Dec 28;18:373. doi: 10.1186/s12882-017-0793-1 (PMC5746009; doi:10.1186/s12882-017-0793-1)

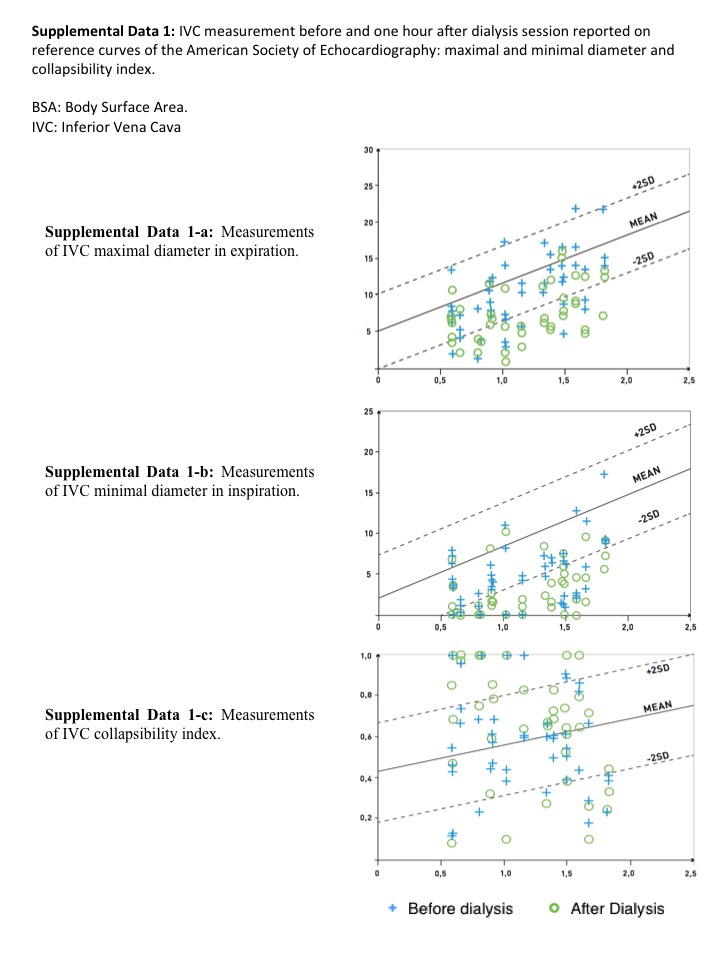

Supplement: Additional file 1: — IVC measurement before and one hour after dialysis session reported reference curves of the American Society of Echocardiography: maximal and minimal diameter and collapsibility index. (JPEG 101 kb) [file 12882_2017_793_MOESM1_ESM.jpg]
